# The Role of Monocytes and Macrophages in Homeostasis and Disease and Novel Avenues for Putative Treatments

**DOI:** 10.3390/ijms22094927

**Published:** 2021-05-06

**Authors:** Malgorzata Kloc, Jacek Z. Kubiak

**Affiliations:** 1 Houston Methodist Research Institute, Houston, TX 77030, USA; 2 Department of Surgery, Houston Methodist Hospital, Houston, TX 77030, USA; 3 M.D. Anderson Cancer Center, Department of Genetics, University of Texas, Houston, TX 77030, USA; 4 Department of Regenerative Medicine and Cell Biology, Military Institute of Hygiene and Epidemiology (WIHE), 01-163 Warsaw, Poland; 5 Dynamics and Mechanics of Epithelia Group, Faculty of Medicine, Institute of Genetics and Development of Rennes (IGDR), University of Rennes, CNRS, UMR 6290, 35000 Rennes, France

Macrophages were discovered in the 19th century by Ukrainian biologist Élie Metchnikoff who worked in Ukraine, Russia, and France [1,2]. He discovered the process of phagocytosis, i.e., the ability to devour (phagocyte) microorganisms and cell debris, and understood that it plays a major role in the immune system. In 1908, he received the Noble prize for his research on immunity [3]. Phagocytosis was the principal function attributed to macrophages for many decades. This ability that evolved over 500 million years ago in the unicellular amebae allowed macrophages to be the first responders of the immune system against invading pathogens and the leading debris cleaners during wound healing. Over the years, we have learned that phagocytosis is only a tiny fraction of the abilities of macrophage and that macrophages are not only highly complex and multifunctional but also incredibly malleable, and depending on the need and environment, they can switch properties, phenotypes, and functions or curb their voracious instinct to become tolerant (and even collaborative) to the beneficial symbiotic microorganisms inhabiting the host [4,5]. Moreover, by presenting antigens and secreting various cytokines and factors, macrophages communicate with other immune cells, sculpt the overall response of the innate and adaptive immune system, and control long-term (chronic) rejection of transplanted organs [6,7]. Additionally, by exchanging molecules and organelles such as mitochondria with the immune cells and stem cells, macrophages shape the regenerative responses [8,9].

One myth that has been prevailing for the last 50 years pertains to the origin of macrophages. In 1968, van Furth and Cohn proposed that all macrophages derive from the circulating blood monocytes, and only a decade ago was it definitively accepted that adult organism’s macrophages have a dual origin: not only from the monocytes but also from the embryonic yolk sack [10]. It is now well established that all tissues and organs have highly specialized populations of resident macrophages, which derive from the embryonic yolk sack, and perform tissue/organ-specific functions. In the last few decades, we also learned that the tumor-associated macrophages (TAMs), can fuse with cancer cells and bestow on them motility necessary for metastasis [11,12,13].

Considering these multifaceted functions of macrophages, it is not surprising that they are affected by various drugs and compounds used in the clinic, and that they become a favorite target for the development of novel therapies for many human diseases [14,15].

In this issue, we present an overview of the current research trends concerning macrophages and their newly discovered properties and functions. We focused this Special Issue on both human and animal macrophages because different animal models are very helpful in the understanding of novel roles of these immune cells and in the development of new clinically applicable therapies. In this issue, Tsuji et al. described the effect of antipyretic drug acetaminophen (APAP, which causes liver injury when overdosed) on M1/M2 macrophages in the rat model [16]. They provide a new perspective on hepato-pathogenesis and direct novel avenues for the development of therapeutic strategies. Bhunyakarnjanarat and colleagues delineated the effects of a common analgesic, NSAIDs, on macrophages and correlation with the gastrointestinal permeability defect (gut leakage) and lupus [17]. Their research strengthens the possibility that lupus disease activation may be related to the NSAID-enteropathy-induced gut leakage. In the same issue, Barczak et al. described the effect of a new silicate cement mineral, trioxide aggregate (MTA Repair HP), on the macrophage-related inflammation processes of the tooth and periodontal tissues [18]. The material studied here is used in the regeneration of the pulp–dentin complex, treatments of teeth perforations, and periapical tissue. This study shows that MTA Repair HP does not induce macrophage activation or increase the MMP-2 and MMP-9 metalloproteinases. Thus, they concluded that this new compound does not increase the inflammatory response in the analyzed population of monocytes/macrophages and does not affect the dentin regeneration in which MMP-2 and MMP-9 are normally involved. The participation of macrophages/foam cells in the development of atherosclerosis was described by Yashima et al. [19]. Advanced glycation end products (AGEs) localize to macrophage-derived foam cells within atherosclerotic lesions, which is associated with a higher risk of atherosclerotic cardiovascular disease under diabetic conditions. This article suggests that AGEs may stimulate ox-LDL uptake by macrophages through the Cdk5–CD36 pathway via RAGE-mediated oxidative stress. The role of macrophages in atherosclerosis is an important issue in current medicine. It is also reviewed, with special focus on the RhoA pathway, in the Kloc et al. article [20]. In this review article, we describe how the engulfment of oxidized and acetylated low-density lipoproteins (oxLDLs and AcLDLs) and minimally modified LDLs (mmLDLs) by macrophages, recruited into the vessel wall or residing in the vessel wall intima, leads to the formation of lipid laden macrophages (foam cells). We also pay special attention to the small GTPase RhoA and its downstream effectors because they regulate the influx of macrophages and control the macrophage phenotype, inflammatory or anti-inflammatory, involved in the development, progression, and stabilization of the atherosclerotic plaque. RhoA-interfering drugs promise novel therapeutic approaches against atherosclerosis. Finally, another article from Kloc et al. laboratories describes how the inhibition of the macrophage influx into transplanted organs by inhibition of the RhoA pathway effector ROCK kinase prevents the development of the long-term (chronic) rejection of transplanted organs. We also summarize our present knowledge on the macrophage response to microorganisms and transplanted organs and suggest potential novel therapeutic approaches to fight microbial infections and rejection of transplanted organs [21]. This series of articles gathered in our Special Issue presents an overview of the recent trends in the research on the role of macrophages/monocytes in tissue homeostasis and disease, including potential new avenues for the development of clinical therapies.

## Data Availability

Not applicable.

## References

[1] Silverstein A.M. (2011). Ilya Metchnikoff, the phagocytic theory, and how things often work in science. J. Leukoc. Biol..

[2] Merien F. (2016). A Journey with Elie Metchnikoff: From Innate Cell Mechanisms in Infectious Diseases to Quantum Biology. Front. Public Health.

[3] Kaufmann S.H.E. (2008). Immunology’s foundation: The 100-year anniversary of the Nobel Prize to Paul Ehrlich and Elie Metchnikoff. Nat. Immunol..

[4] Kloc M., Uosef A., Elshawwaf M., Abdelshafy A.A.A., Elsaid K.M.K., Kubiak J.Z., Ghobrial R.M. (2020). The Macrophages and Intestinal Symbiosis. Results Probl. Cell Differ..

[5] Alpdundar Bulut E., Bayyurt Kocabas B., Yazar V., Aykut G., Guler U., Salih B., Surucu Yilmaz N., Ayanoglu I.C., Polat M.M., Akcali K.C. (2020). Human Gut Commensal Membrane Vesicles Modulate Inflammation by Generating M2-like Macrophages and Myeloid-Derived Suppressor Cells. J. Immunol..

[6] Zhao Y., Chen S., Lan P., Wu C., Dou Y., Xiao X., Zhang Z., Minze L., He X., Chen W. (2018). Macrophage subpopulations and their impact on chronic allograft rejection versus graft acceptance in a mouse heart transplant model. Am. J. Transplant..

[7] Locati M., Curtale G. (2020). Mantovani A Diversity, Mechanisms, and Significance of Macrophage Plasticity. Annu. Rev. Pathol..

[8] Jackson M.V., Morrison T.J., Doherty D.F., McAuley D.F., Matthay M.A., Kissenpfennig A., O’Kane C.M., Krasnodembskaya A.D. (2016). Mitochondrial Transfer via Tunneling Nanotubes is an Important Mechanism by Which Mesenchymal Stem Cells Enhance Macrophage Phagocytosis in the In Vitro and In Vivo Models of ARDS. Stem Cells.

[9] Kloc M., Uosef A., Leśniak M., Kubiak J.Z., Ghobrial R.M. (2020). Reciprocal interactions between mesenchymal stem cells and macrophages. Int. J. Dev. Biol..

[10] Epelman S., Lavine K.J., Randolph G.J. (2014). Origin and functions of tissue macrophages. Immunity.

[11] Kloc M., Li X.C., Ghobrial R.M. (2016). Are Macrophages Responsible for Cancer Metastasis?. J. Immunobiol..

[12] Sanchez L.R., Borriello L., Entenberg D., Condeelis J.S., Oktay M.H., Karagiannis G.S. (2019). The emerging roles of macrophages in cancer metastasis and response to chemotherapy. J. Leukoc. Biol..

[13] McDonald M.K., Tian Y., Qureshi R.A., Gormley M., Ertel A., Gao R., Aradillas Lopez E., Alexander G.M., Sacan A., Fortina P. (2014). Functional significance of macrophage-derived exosomes in inflammation and pain. Pain.

[14] Ponzoni M., Pastorino F., Di Paolo D., Perri P., Brignole C. (2018). Targeting Macrophages as a Potential Therapeutic Intervention: Impact on Inflammatory Diseases and Cancer. Int. J. Mol. Sci..

[15] Duan Z., Luo Y. (2021). Targeting macrophages in cancer immunotherapy. Signal Transduct. Target. Ther..

[16] Tsuji Y., Kuramochi M., Golbar H.M., Izawa T., Kuwamura M., Yamate J. (2020). Acetaminophen-Induced Rat Hepatotoxicity Based on M1/M2-Macrophage Polarization, in Possible Relation to Damage-Associated Molecular Patterns and Autophagy. Int. J. Mol. Sci..

[17] Bhunyakarnjanarat T., Udompornpitak K., Saisorn W., Chantraprapawat B., Visitchanakun P., Dang C.P., Issara-Amphorn J., Leelahavanichkul A. (2021). Prominent Indomethacin-Induced Enteropathy in FcgRIIB-Deficient lupus Mice: An Impact of Macrophage Responses and Immune Depositionin Gut. Int. J. Mol. Sci..

[18] Barczak K., Palczewska-Komsa M., Lipski M., Chlubek D., Buczkowska-Radlińska J., Baranowska-Bosiacka I. (2021). The Influence of New Silicate Cement Mineral Trioxide Aggregate (MTA Repair HP) on Metalloproteinase MMP-2 and MMP-9 Expression in Cultured THP-1 Macrophages. Int. J. Mol. Sci..

[19] Yashima H., Terasaki M., Sotokawauchi A., Matsui T., Mori Y., Saito T., Osaka N., Kushima H., Hiromura M., Ohara M. (2020). AGE-RAGE Axis Stimulates Oxidized LDL Uptake into Macrophages through Cyclin-Dependent Kinase 5-CD36 Pathway via Oxidative Stress Generation. Int. J. Mol. Sci..

[20] Kloc M., Uosef A., Kubiak J.Z., Ghobrial R.M. (2021). Role of Macrophages and RhoA Pathway in Atherosclerosis. Int. J. Mol. Sci..

[21] Kloc M., Uosef A., Kubiak J.Z., Ghobrial R.M. (2020). Macrophage proinflammatory responses to microorganisms and transplanted organs. Int. J. Mol. Sci..

